# Surgical and Oncologic Outcome following Sacrectomy for Primary Malignant Bone Tumors and Locally Recurrent Rectal Cancer

**DOI:** 10.3390/cancers16132334

**Published:** 2024-06-26

**Authors:** Anne Weidlich, Klaus-Dieter Schaser, Jürgen Weitz, Johanna Kirchberg, Johannes Fritzmann, Christian Reeps, Philipp Schwabe, Ingo Melcher, Alexander Disch, Adrian Dragu, Doreen Winkler, Elisabeth Mehnert, Hagen Fritzsche

**Affiliations:** 1 University Center for Orthopedics, Trauma Surgery and Plastic Surgery, Sarcoma Center at the National Center for Tumor Diseases (NCT/UCC), University Hospital Carl Gustav Carus Dresden, 01307 Dresden, Germany; klaus-dieter.schaser@ukdd.de (K.-D.S.); alexander.disch@ukdd.de (A.D.); adrian.dragu@ukdd.de (A.D.); doreen.winkler@ukdd.de (D.W.); elisabeth.mehnert@ukdd.de (E.M.); hagen.fritzsche@ukdd.de (H.F.); 2 Department of Visceral, Thoracic and Vascular Surgery, incl. Division of Vascular and Endovascular Surgery, Sarcoma Center at the National Center for Tumor Diseases (NCT/UCC), University Hospital Carl Gustav Carus Dresden, 01307 Dresden, Germany; juergen.weitz@ukdd.de (J.W.); johanna.kirchberg@ukdd.de (J.K.); johannes.fritzmann@ukdd.de (J.F.); christian.reeps@ukdd.de (C.R.); 3 Department for Trauma and Orthopedic Surgery, Center for Musculoskeletal Tumor Medicine, Vivantes Hospital Spandau, 13585 Berlin, Germany; philipp.schwabe@vivantes.de (P.S.); ingo.melcher@vivantes.de (I.M.)

**Keywords:** sacrectomy, navigation, primary malignant tumor, chordoma, sarcoma, sacrum, colorectal carcinoma, rectal cancer, surgical margins, oncologic outcome

## Abstract

**Simple Summary:**

Sacrectomy represents a radical indication for bone sarcomas (e.g., osteosarcoma or chondrosarcoma) and chordomas, as well as selected carcinomas with invasion of the sacrum. Extralesional en bloc excision is surgically demanding and associated with resection-induced neurologic deficits and risks. Due to the low incidence of bone sarcomas, the rare localization in the sacrum and the complexity of the surgical procedure, studies reporting on the oncological outcome and corresponding complications in larger patient numbers are rare. The aim was to describe the oncosurgical management and the complication profile and to analyze our own treatment results after partial/total sacrectomy, with attention paid to a possible benefit by using intraoperative 3D navigation. There was a significant difference in progression-free and metastasis-free survival between sarcoma, chordoma and carcinoma patients. Complications were common, but no independently influencing causative factors could be identified. Although there was a subjective impression of improved intraoperative 3D orientation and easier identification of resection planes, the use of navigation did not significantly influence resection status or oncological patient outcome.

**Abstract:**

Introduction: Bone sarcoma or direct pelvic carcinoma invasion of the sacrum represent indications for partial or total sacrectomy. The aim was to describe the oncosurgical management and complication profile and to analyze our own outcome results following sacrectomy. Methods: In a retrospective analysis, 27 patients (n = 8/10/9 sarcoma/chordoma/locally recurrent rectal cancer (LRRC)) were included. There was total sacrectomy in 9 (incl. combined L5 en bloc spondylectomy in 2), partial in 10 and hemisacrectomy in 8 patients. In 12 patients, resection was navigation-assisted. For reconstruction, an omentoplasty, VRAM-flap or spinopelvic fixation was performed in 20, 10 and 13 patients, respectively. Results: With a median follow-up (FU) of 15 months, the FU rate was 93%. R0-resection was seen in 81.5% (no significant difference using navigation), and 81.5% of patients suffered from one or more minor-to-moderate complications (especially wound-healing disorders/infection). The median overall survival was 70 months. Local recurrence occurred in 20%, while 44% developed metastases and five patients died of disease. Conclusions: Resection of sacral tumors is challenging and associated with a high complication profile. Interdisciplinary cooperation with visceral/vascular and plastic surgery is essential. In chordoma patients, systemic tumor control is favorable compared to LRRC and sarcomas. Navigation offers gain in intraoperative orientation, even if there currently seems to be no oncological benefit. Complete surgical resection offers long-term survival to patients undergoing sacrectomy for a variety of complex diseases.

## 1. Introduction

Primary malignant bone tumors, particularly chordomas, chondrosarcomas and osteosarcomas, are some of the extremely rare cancers with an incidence between 0.5 and 1/1,000,000 [1,2,3,4]. Localization to the sacrum is even rarer, as only 1–3.5% of all primary malignant bone tumors and 7% of all spinal malignancies show sacral manifestation. In contrast, sacral involvement in the direct local growth of locally recurrent colorectal carcinoma) is more frequently observed as 6–38% of rectal cancers recur, of which 25–50% recurrence is limited to the pelvis [5,6].

Typically, patients with sacral malignancies are either incidentally diagnosed or present with large and advanced intrapelvic tumors. Due to long asymptomatic expansive intrapelvic tumor growth, initial symptoms are mild, and patients become aware of the tumor not before they develop signs for tumor-induced visceral dysfunction or neurovascular compression. Decision making for the therapeutic management of sacral tumors, including indication for neoadjuvant therapy, should be guided by overall expected morbidity of the procedures, tumor-specific, patient-specific and procedure-related characteristics. Most of all underlying tumor biology/radioresistance, deliverability of radiotherapy, response to chemotherapy and involvement of neurovascular structures, as well as age, performance status, comorbidities and options for spinopelvic fixations with possibly also plastic coverage, are guiding the respective and specific indications. 

Intralesional resection without adequate surgical margins may lead to a higher rate of local recurrence [7,8,9,10]. Hence, either en bloc or maximum gross total resection with high-dose radiotherapy has been demonstrated to increase the disease-free survival period in patients harboring various primary sacral neoplasms that are unresponsive to chemotherapy and conventional radiotherapy [8,9,10]. Despite the obvious advantages for disease-free survival, en bloc resection and spinopelvic reconstruction for sacral tumors pose a unique challenge for the surgeon in view of the complex anatomical and biomechanical relationships that govern the spinopelvic junction. Although partial, distal sacrectomy is usually well tolerated, patients with either extensive intrapelvic expansion or tumor growth higher than the S2-Segment require high or total sacrectomy that typically not only requires the sacrifice of S1-/S2-nerve roots with consecutive sensomotor dysfunction but also results in vertical and rotational pelvic instability necessitating spinopelvic fixation to allow for effective pain control and improved ambulation [11,12,13].

While different surgical resection techniques and diverse reconstruction options are well described [9,14,15,16,17,18], the postoperative early and late complication rates continue to be relatively high, and their management requires demanding interdisciplinary management [1,19]. Due to the low incidence of certain tumor entities and their even rare localization in the sacrum, data for surgical resection technique, oncological outcome and complication profile have limited availability. 

Therefore, this study aimed to retrospectively describe the surgical management (including the use of 3D navigation), the oncologic outcome and the complications after total or partial sacrectomy in patients with primary malignant bone tumors of the sacrum or solitary sacral involvement due to locally recurrent rectal carcinomas.

## 2. Materials and Methods

In a retrospective, multicenter study, from September 2015 to December 2023, all patients in the University Center for Orthopaedics, Trauma and Plastic Surgery of the University Hospital Dresden, in the Department of Visceral, Thoracic and Vascular Surgery of the University Hospital Dresden, and in the Department for Musculoskeletal Surgery of the Vivantes Hospital Berlin–Spandau who underwent either complete, partial or hemisacrectomy due to a primary malignant bone tumor or locally recurrent rectal cancer of the sacrum were included. Patients with metastatic disease or without oncological indication for sacrectomy were excluded. Patients’ informed consent and approval by the local ethics committee was obtained (institutional review board BO-EK-235042021).

Demographic and clinical–pathological data, such as gender, age at diagnosis, type of the tumor and resection status after surgery, metastases, local recurrence, and neoadjuvant or adjuvant therapies, were analyzed. The primary endpoints of this study were to determine the overall, disease-free, recurrence-free and metastases-free survival after sacrectomy. The secondary endpoints were the rate of complications, especially the rate of infection and wound complications requiring surgical revision, and the assessment of ambulatory status.

### 2.1. Surgical Technique

Patients with a purely posterior and combined anterior–posterior approach were included. Decision making for combined approaches was made based on the level of planned sacrectomy; intrapelvic paraosseous tumor growth; the involvement of visceral/urogenital structures, lumbar spine, lateral pelvic sidewall, and/or the sciatic notch, as well as encasement of iliac vessels. All patients with total and high (above the third sacral body) sacrectomies and/or suspicion of vascular involvement in angiographic CT/MRI-scans (independent from sacral resection level) were treated by combined approaches, as a two-stage procedure with initial anterior surgery, followed by posterior surgery with tumor resection on the next day. In these patients, ureteral stents were placed the day prior to anterior surgery.

**Figure 1:**
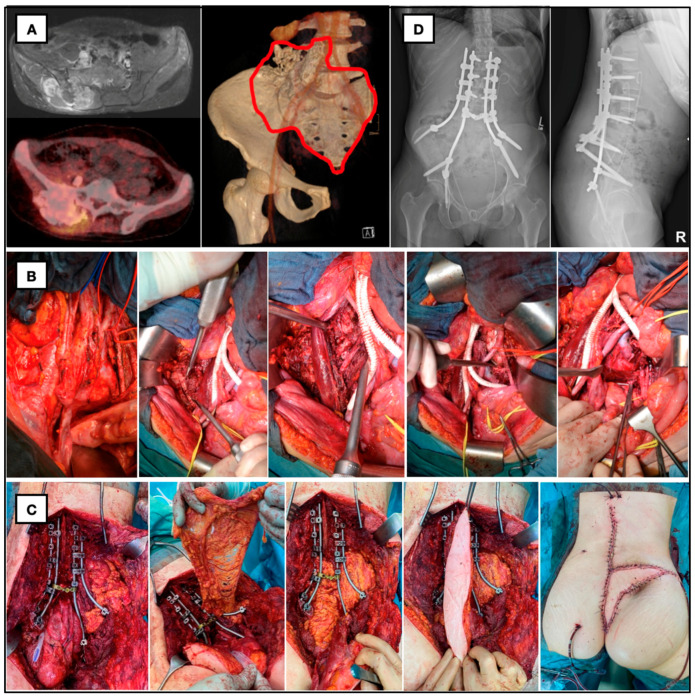
Twenty-eight-year-old female patient with giant ilio-lumbosacral osteosarcoma. (**A**) Preop. MRI, PET and CT with planned resection margins. (**B**) Anterior approach with aortic bifurcation, vascular replacement of vena cava, osteotomy of the right iliac wing and discectomy of L4/L5. (**C**) En bloc resection of os sacrum and right iliac wing, spinopelvic fixation with double-rod construction, preparation of omentum majus for death space filling and defect reconstruction with VRAM-flap. (**D**) Postop. X-ray with iliolumbar/spinopelvic fixation.

*Anterior approach*: Laparotomy was performed through a lower midline incision, and the left colon was mobilized, followed by vascular exposure, including the lower aorta, the common and external iliac arteries, and veins on both sides. Circumferential exposure of the arteries was usually necessary to expose and control the veins. Branches of the internal iliac artery and vein supplying or adhering to the tumor were ligated; however, if oncologically adequate, special attention was paid to the protection of the superior gluteal artery for the preservation of gluteal muscle perfusion. In some patients, ligation of the internal iliac vein was necessary due to massive tumor growth and prevention of venous distension and bleeding. Anterior dissection was performed according to tumor extent; in patients with recurrent rectal cancer this typically involved partial or total pelvic exenteration. The pelvic sidewall dissection was individualized to achieve complete tumor resection. L5 and S1 nerve roots, as well as the obturator nerve, were typically dissected and preserved if possible. Using either CT-based navigation or intraoperative fluoroscopy control, the sacral level of either horizontal or vertical (hemisacrectomy) transection was determined, and monocortical osteotomies with straight or angulated chisels were performed (Figure 1). Depending on the sacral resection level, vertical lateral transections of either the ilium or along the oblique course of the sacroiliac joints were carried out in the same way, without injury to the sacral articular surface, leaving the sacral compartment intact. For total sacrectomies, the anterior longitudinal ligament was dissected at the level of the promontorium, and the intervertebral disc L5/S1 was removed using rongeurs. In all patients with high or total sacrectomies, pedicled omentum majus was transferred into the pelvis (omentoplasty) for dead-space filling and further reduction of postoperative deep infections. During anterior surgery, also the vertical rectus abdominis myocutaneous (VRAM) flap was elevated for subsequent perineal–gluteal reconstruction in all patients that received neoadjuvant radiotherapy and/or required extended gluteal soft tissue reconstruction due to massive posterior tumor extension [20]. All patients with combined approaches for locally recurrent rectal carcinoma had already had an end-colostomy due to abdominoperineal resection in the past or received the colostomy as part of the procedure. All other patients requiring anterior approaches first received a protective temporary loop ileostomy or transverse colostomy to divert feces and reduce risk for postoperative infection of posterior wound. Reduction of ostomy with reanastomosis of the ileum or colon was performed after completed wound healing, but not before 6 weeks postoperatively. If anus sphincter function was not sufficient and did not recover over time, a permanent colostomy was performed. At the end of the anterior procedure, a sterile plastic sheet was placed in the presacral plane between the vessels and the posterior sacral tumor to protect them from possible injury by chisel osteotomy during posterior surgery.

*Posterior approach*: With the patient in a prone and modified lithotomy position posterior, midline incision extending to the lower lumbar spine (depending on necessity of number of lumbar levels for spinopelvic fixation) to the coccyx was used. Gluteus maximus muscles were detached from the sacrum, sciatic notches and nerves. The posterior iliac crests were exposed bilaterally. The sacrotuberous and -spinous ligaments, as well as the lower sacral nerve roots, were transected to gain access to the presacral pelvic space. Depending on the sacral level of resection either sacral or lumbar (L5), laminectomy was performed; lowest sacral nerve roots to be preserved were identified; and dural sac ligation and transection, followed by double sutures, were performed. In hemisacrectomy, the dural sac was preserved, and only unilateral roots were ligated and transected. The level of horizontal sacral osteotomy or the disc space L5/S1 (in total sacrectomy), as well as the median (hemisacrectomy) and lateral vertical osteotomies, was identified using either navigation or fluoroscopic control and divided by a broad chisel, aiming at and connecting to the anterior osteotomy. For navigation-assisted resections and intraoperative 3D navigation, an electronic navigation system (Brainlab, Munich, Germany) with a computer workstation and coupled to a reference base fixed to the patient and infrared camera system was used. Using preoperatively fused different MRI and low slice thickness (0.6–1 mm) CT (also possible with PET) datasets, landmark and surface matching with registration of instruments (pointers and chisels) were reached. In patients who underwent combined approaches, a CT-scan was made after anterior surgery that was used for posterior navigation the next day and allowed us to visualize and navigate to the unicortical osteotomies already performed from the anterior.

After resection, the protective plastic sheet, put in from the anterior, was removed, and both the specimen and the cavity were inspected. Then, multiple intraoperative frozen sections from the bony and soft tissue margins of the patient were sent to the pathologist for immediate assessment of surgical margins. Any questionable positive margins were re-resected. Iliolumbar/spinopelvic fixation and flap reconstruction were not used in all patients. All patients with total sacrectomies and high sacral resections, leaving only a narrow and small S1 segment with raising concerns regarding stability, were treated by spinopelvic fixation, using instrumentation with polyaxial transpedicular screw and double-rod constructs connected to long and thick (9–10 mm) iliac screws (Figure 1). Soft tissue reconstruction was realized either by means of the anteriorly prepared pedicled omentoplasty and myocutaneous rectus (VRAM-) flap (Figure 1) or suturing the remaining gluteus muscles together on both sides after prophylactic topical application of the vancomycin [21].

### 2.2. Aftercare and Restaging

Immediately postoperatively, pain management and efficient analgesia were ensured, and mobilization, including standing and walking, was allowed according to the tolerated pain levels. The adjuvant therapy was performed according to the guidelines for the individual underlying malignancy in accordance with the tumor board decision and included protocol-based neoadjuvant chemotherapy, immunotherapy, and/or radiotherapy. After the completion of treatment, patients were followed up regularly as part of the guideline-based tumor follow-up (sarcoma: clinical and imaging examinations by local MRI and CT of the thorax every 3 months for 2 years, semiannually from the 3rd year, and annually from the 6–10th year; colorectal carcinomas: CT thorax/abdomen every 6 months for 2 years and then annually until the 5th year). 

### 2.3. Statistics

The IBM SPSS Statistics program was used for the analyses. The social–demographic and clinicopathological patient characteristics were analyzed descriptively by means of median, minimum and maximum. By making cross-tabulations, complications that occurred were analyzed as categorized parameters and compared using the chi-square test. Risk factors were considered as independent variables. Survival was estimated according to the Kaplan–Meier method and compared using the log-rank test. In this study, unless otherwise specified, the significance level was 5% on both sides, or a 95% CI was presented.

## 3. Results

### 3.1. Clinical Characteristics

The retrospective, multicenter study included 27 patients who underwent surgery from September 2015 to December 2023 at the University Center for Orthopaedics, Trauma and Plastic Surgery of the University Hospital Dresden (*n* = 13), at the Department for Visceral, Thoracic and Vascular Surgery of the University Hospital Dresden (*n* = 9) or at the Department for Trauma and Orthopedic Surgery of the Vivantes Hospital Spandau (*n* = 5). Of the 27 patients (female/male: 13/14), the mean age at the time of surgery was 58 (16–86) years. Primary malignant tumor was present in 67% of patients (chordoma, osteosarcoma, rhabdomyosarcoma, chondrosarcoma, pleomorphic sarcoma and liposarcoma), and 33% of patients showed infiltration of the sacrum by locally recurrent rectal/colon carcinoma (Table 1). Neoadjuvant systemic therapy was performed in 55.6% (48.1% chemotherapy and 22.2% immunotherapy), while 29.6% of the patients were treated with neoadjuvant radiation. In one-third of all patients, purely posterior approaches were performed. For all other patients, sequentially combined anterior–posterior approaches were used, which is very much in line with the finding that, in 63% of the patients, preoperative CT/MR-imaging showed an extensive presacral tumor extension. Total sacrectomy was performed in 33.3% of patients, partial high (above the S3 segment) and low (at or below S3 segment) in 18.5% and 18.5%, respectively. While total hemisacrectomy was performed in 22.2% of patients, the hemisacrum was partially resected in 7.5% of patients, with additional partial pelvic or SI-joint resection in 26%. In half of all patients (all total and hemi-, as well as some high, sacrectomies), spinopelvic reconstruction using screw–rod constructs was performed. In more than three-quarters of all patients, an indication for a temporary ileo-/colostomy was seen, and intrapelvic omentum majus transfer (vascular pedicled omentoplasty) for dead-space filling of the cavity was performed in 74% of all patients. With a similar frequency of about one-third of all patients who received neoadjuvant radiotherapy, also a myocutaneous (VRAM) flap was prepared (in 37%) for plastic reconstruction on the first day of surgery, together with the omentum majus transferred posteriorly after completed resection at the second day of surgery to fill and cover the defect. In 12 patients (44.4%), resection was performed with image-guidance and navigation, using a fusion of preoperative CT, MRI and, if available, PET datasets. In 81.5% of all patients, the resection was performed completely with Enneking-appropriate clean surgical margins (R0 resection); in 18.5%, the resection margin could not be assessed histologically (Rx resection), or a microscopic tumor remnant (R1 resection, Enneking-inadequate) was seen (Table 1).

### 3.2. Survival Analysis

Of the 27 patients included, current follow-up data were available for 25 patients at the end of the follow-up period, resulting in a follow-up rate of 93%. The median follow-up of all 27 patients was 27 (1–100) months. Two foreign patients were lost to follow-up after 1 and 10 months, respectively, as no contact could be established despite repeated attempts.

At the end of the follow-up period, 18 patients (67%) were alive; 6 patients (22%) died of metastatic disease at 3, 4, 14, 20, 25 and 27 months postoperatively; and 1 (3.7%) patient without any evidence of disease died 5 months after multiple revision surgeries due to multiorgan failure. Therefore, according to Kaplan–Meier, the mean disease-specific survival was 77 months (95% CI [62; 93]). Interestingly, disease-specific survival following sacrectomy in sarcoma patients (without chordoma patients (48 months)) (34 months (95% CI [21; 47]) did not differ significantly (*p* = 0.887) from patients with locally recurrent carcinoma (68 months (95% CI [39; 97]). The Kaplan–Meier 1- and 2-year overall survival rates are 89% and 80%, respectively. Again, no significant differences in 1- and 2-year overall survival rates were seen for sarcoma and carcinoma patients.

The median progression-free survival (metastasis or local recurrence), according to Kaplan–Meier, was 38 months (95% CI [0; 38]) for all, with significant longer intervals in chordoma patients compared to sarcoma and carcinoma patients (*p* = 0.003). Local recurrences occurred in seven patients after a median time of 9 (7–38) months. Eleven patients were diagnosed with distant metastases after median 8 (1–38) months, while seven of these patients showed both distant metastases and local recurrence. At the time of the last follow-up, 13 patients (48%) were without evidence of disease (Table 2). 

The mean metastases-free and average recurrence-free survival were 46 months (95% CI [29; 64]) and 69 months (95% CI [51; 88]), respectively. Notably, recurrence-free and metastases-free survival were significantly longer in chordoma patients than in sarcoma or carcinoma patients (*p* = 0.003 and *p* = 0.024, respectively).

**Figure 2:**
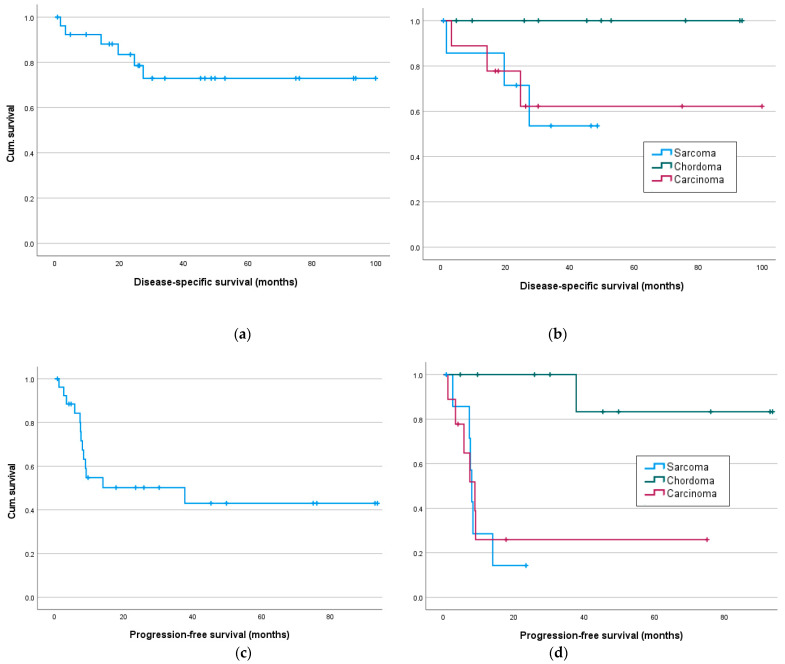
Disease-specific and progression-free survival in months: (**a**) disease-specific-survival for all and (**b**) for sarcoma, chordoma and carcinoma patients; progression-free survival for all (**c**) and (**d**) sarcoma, chordoma and carcinoma patients.

In disease-specific survival, the log-rank tests showed no significant differences (Figure 2b and Table 3) between sarcoma, chordoma and carcinoma patients. But as expected, the occurrence of metastases in the follow-up period significantly affected the disease-specific survival (χ^2^(1) = 5.71, *p* = 0.017).

**Figure 3:**
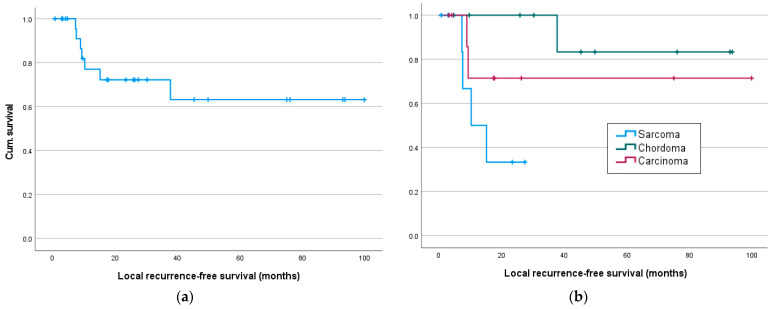

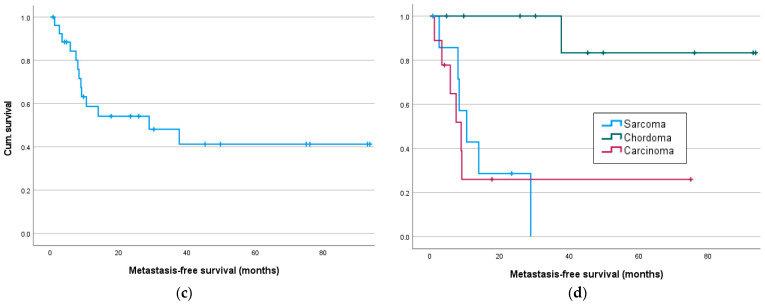
Local recurrence- and metastasis-free survival in months: (**a**) Local recurrence-free survival for all and (**b**) for sarcoma, chordoma and carcinoma patients; (**c**) metastasis-free survival for all and (**d**) sarcoma, chordoma and carcinoma patients.

Significant results are seen in metastasis-free survival, as a tumor entity was associated with significantly different survival rates, especially with better survival of chordoma patients (sarcoma/chordoma/carcinoma) (χ^2^(1) = 11.95, *p* = 0.003, Figure 3d), especially between sarcoma/chordoma (χ^2^(1) = 13.54, *p* < 0.001) and chordoma/carcinoma (χ^2^(1) = 8.82, *p* = 0.003). Significant differences in survival were also demonstrated for neoadjuvant chemotherapy, as well as immunotherapy, with worse survival of patients with neoadjuvant systemic therapy (Table 3).

Recurrence-free survival (Figure 3a) was only significantly affected by the resection status or adjuvant radiation but was significantly better in chordoma patients compared to sarcoma patients (sarcoma/chordoma (χ^2^(1) = 7.81, *p* = 0.005; Table 3).

A Cox regression analysis was not performed because of the many variables and the small number of cases.

### 3.3. Complications

In 22 patients (81.5%), complications occurred postoperatively, while in all patients with complications, at least one or more revision procedures were necessary (Table 4). The most frequent postoperative complication was a wound-healing disorder or infection (*n* = 17, 63%, in 74% even with stoma), which led to sepsis in three patients (11%), followed by hematoma/postoperative bleeding (*n* = 6, 22%) and a postoperative hardware failure with rod/screw breakage or dislocation (*n* = 6, 22%). Secondary postoperative hardware failure (rod/screw/connector dislocation/loosening and later breakage) was found in four and two patients after a mean duration of 4 and 19, respectively. All required reinstrumentation with screw/rod exchange—the two patients with screw malposition or loosening of the screw connector already during their initial hospitalization (Table 4). Two patients suffered from an insufficiency fracture of the remaining S1-body segment after partial high sacrectomy or partial hemisacrectomy below S1 and were treated with a hexacortical sacroliliac (SI) screw fixation.

All complications were analyzed and screened for possible influencing factors using cross-tables and a chi-square test: gender, age over 60 years, neoadjuvant systemic or radiotherapy, adjuvant radiotherapy, resection level (partial/total), approach, use of navigation, spinopelvic fixation, omentoplasty, VRAM flap and stoma (Table 5). Regarding infections and wound-healing disorders, the univariate regression analysis showed significantly fewer deep infections with the use of a VRAM flap and significantly fewer superficial wound-healing disorders with preoperative stoma. Neoadjuvant radiotherapy seemed to be associated with a slight tendency towards an increased risk of wound-healing disorders (Table 5). A multivariate/logistic regression analysis was not performed because of the many variables and the small number of cases.

There was a significant correlation between implant-associated complications and neoadjuvant radiotherapy (χ^2^(1) = 5.08, *p* = 0.024, Fisher’s exact test *p* = 0.044), as well as between thromboembolic events and stoma creation (χ^2^(1) = 7.56, *p* = 0.006, Fisher’s exact test *p* = 0.043). No significant results were shown for any of the other variables tested. However, the clinical relevance of these results is questionable with low case numbers.

## 4. Discussion

### 4.1. Clinical Characteristics, Surgical Resection and Margins

Primary malignant tumors and solitary metastases in the sacral region are rare, which becomes apparent by the low number case series [13,22,23]. In our study of 27 patients, chordomas (37%), followed by osteosarcomas (11%), was the most common in the sarcoma group, followed by colorectal carcinomas (33%) in equally high numbers. This is consistent with some other studies, which also have observed chordomas and osteosarcomas as the most common tumor entities [1,9,12,15,24]. Regarding sacral metastases, studies most frequently identified renal cell carcinoma [10,13], lung carcinoma [10], and breast carcinoma [24] as underlying tumor biologies. Additionally, advanced presacral growth of colorectal carcinomas may cause infiltration of the sacrum, especially for patients with extraluminal local recurrence after potentially curative resection showing an incidence of pelvic/sacral invasion of 5–35% [10,13,25,26].

Resection of sacral tumors is very challenging because of complex intrapelvic/sacral anatomy with close proximity to important neurovascular, visceral and urogenital anatomic structures. For sarcomas/chordomas and locally recurrent rectal carcinomas, the principal goal is a wide local en bloc and margin-free (R0-) resection. For this, appropriate surgical approaches should be chosen that allow surgeons to achieve an extralesional en bloc resection while having maximum control to protect or even dissect/ligate iliac vessels, nerve roots and organs [14,15]. 

Depending on the individual experience and practice, there is a general consensus that sacral tumor involvement below S2/3 segments (except massive intrapelvic tumor expansion/growth) can be managed by all-posterior excision via a posterior approach in cases without infiltration of intrapelvic organs. In distal sacrectomies, digital control of the anterior aspect of the distal sacrum by the surgeon’s finger inserted posterior or lateral via the sciatic notch allows for acceptable detachment of presacral adhesions and the control of the osteotomy [27,28,29]. The risks of the all-posterior approach include less control over the anterior branches of the iliac vessels and increased risk for uncontrolled bleeding. As posterior-only approaches, no anterior release and preparation of peritumoral adhesions can be expected, there may also be a higher risk for a decreased rate of margin-free resections. In patients with anterior expansive tumor growth involving the S3 segment and a consecutive indication for high sacrectomies at the S2 level or even higher, the digital control by the surgeon’s finger is anatomically impossible or may be prevented by the anterior tumor mass. In our study, anterior–posterior combined approaches were performed in 67% of all patients. This may be explained by the relatively high rate of total and high sacrectomies (14 of 27; 52%) and locally recurrent rectal cancer patients. Ozdemir et al. have drawn similar conclusions, preferring a purely posterior approach only for tumors distal to S3 and recommending resection via a combined approach for tumors located further cranially [24]. Pu et al. also favor combined anterior–posterior approaches for total sacrectomies because of the omentum- or rectus-flap plasty requiring anterior preparation via laparotomy [15]. Wang et al. described a mainly posterior approach in 80% of their patients with total, subtotal or hemisacrectomy and 77.5% also undergoing internal fixation [1]. Similarly, Varga et al. also used primarily posterior approaches in 76% [30]. Although involving an additional anterior surgical approach with a certain morbidity, the sequential performance of anterior–posterior approaches has several advantages which maximize patients’ safety. First, mobilization and control of iliac vessels with selective internal iliac branch ligation can be reached. Secondly, nerve root identification, control and, if necessary, sacrifice, as well as ureterolysis, can be easily managed. Thirdly, unicortical osteotomy of the anterior sacral/iliac wall or SI joint proximal and lateral to the tumor can be performed, making the trans-sacral cut and the resection from posterior much easier. Thus, identification of the osteotomy cut when using the CT after anterior surgery for navigation-assisted resection from posterior the day after is facilitated. Fourth, the need for colostomy or VRAM-flap preparation requires an anterior approach first, regardless of the level of sacrectomy. In the experience of our group and others [29], a staged sequential performance of anterior–posterior approaches within 24–48 h shows good results and decreases the individual operation time.

A pedicled tissue transfer for dead-space filling and the prevention of hematoma and wound infection can be managed using omentum majus transfer (omentoplasty) or transpelvic VRAM-flap coverage [15,16,31]. In our study, omentoplasty was performed in 74%, and VRAM flap in 37%. VRAM flap, bilateral gluteal flaps or direct suture by approaching the gluteal muscles are reconstructive options for soft tissue closure [1,15]. In their literature review, Asad et al. described the use of gluteal-based flap in 50%, followed by the VRAM flap (38%) and free latissimus dorsi (5%). Gluteus-based flaps were also used in posterior approaches in the study by Kim et al. (67%). However, patients with total or high sacrectomy and completed sacrifice of gluteal vessels or completed (neo-)adjuvant radiation gluteal flaps or direct soft tissue closure have a high risk for failure. Therefore, in multistage surgeries, they consider the VRAM flap (dissected outside the possible radiation field) as the gold standard for dead space filling [17], very much matching our experiences.

The intraoperative use of 3D navigation has already been discussed in several studies, and possible advantages and disadvantages have been reported. Classic intraoperative imaging with plain radiographs usually provides only static 2D images, with all the known intraoperative restrictions for repeated time-consuming repositioning for biplanar use of the C-arm; poor image quality, particularly for lateral views and in obese patients; and increased radiation exposure [7,8]. Image-guided navigation provides multidimensional positional information and real-time tracking of instruments. In addition, the use of intraoperative 3D imaging is associated with lower radiation exposure than with classical freehand fluoroscopic techniques [9]. Therefore, in our experience, image-guided navigation can be very helpful, especially in non-exposed very complex anatomic regions like the sacrum and pelvis to reduce the risk of neuro-vascular injury, identify and optimize resection margins and minimize both soft tissue trauma and defect size [7,11,12]. 

Real-time intraoperative navigation allows for increased orientation during resections, osteotomies, and instrumentation, as well as feedback regarding the position of the instruments, e.g., chisel tip that would otherwise require additional dissection or extension of surgical approaches [7,8]. In tumor patients with an increased risk of wound-healing disorders and infections, a reduction in soft tissue trauma by using CT-based navigation seems desirable and promising. A disadvantage of CT-based navigation is the sometimes time-consuming and error-prone matching procedure, which can result in a prolonged operation time [32]. However, various studies show that the additional time required for registration and preparation of navigation was only between 17 and 35 min but could decrease with the increasing number of navigations performed [32,33,34,35]. In our study, a meaningful comparison of operation times was not reasonable due to the still-low number and heterogeneity of navigated resections.

Similar to Jeys et al., in our study, matching was performed by surface matching in all patients [36]. Other studies often describe that matching was performed via the fusion of the preoperative dataset with an intraoperative CT scan [11,37,38,39]. Both matching procedures are possible depending on the surgeon’s condition, patient positioning and the individual technical equipment. 

A major goal of the use of intraoperative navigation is more accurate resection margins, particularly in complex anatomical surgical sites. The initial studies demonstrate a benefit of navigation in terms of the increased probability of clean resection margins [11,32,36,38,40]. This is assumed to be the result of improved intraoperative orientation, tumor localization and real-time identification of resection margins. In this context, Laitinen et al. compared navigated and non-navigated pelvic resections, showing a recurrence rate of 22% in the navigated group compared with 50% in the non-navigated group [41]. Other studies also described low local recurrence rates of 13%, 22%, 26%, 22% and 27% [11,36,40,42,43] when using navigation. In our study, no significant differences in the rate of margin-free resections and local recurrence-free survival were found between patients with navigated and non-navigated resections, an outcome that most likely resulted from the relatively low number of navigated cases in our cohort.

Regarding a possible functional relationship between navigated resections and a decreased occurrence of metastases, few data exist. Abraham et al. do not assume any correlation in this aspect; instead, they consider the underlying tumor biology and grading to be the decisive prognostic factors [40]. Also, in our study, there was no correlation in the occurrence of metastases when comparing navigated and non-navigated resections. Bosma reported only a limited use of navigation to achieve clean resection margins in soft tissue. The authors emphasized that inadequate soft tissue resection margins are a negative prognostic factor for local recurrencies, which typically occur at the soft tissue resection margin and not at the osteotomy site [11]. This justified point of criticism, however, can be partly surmounted by using additional MRI datasets as the base for navigation. The fusion of MRI and CT datasets in patients with solid tumors allows for the visualization and navigation of both the intraosseous (osteolysis) and extraosseous soft tissue tumor mass. Moreover, contrast-medium enhancement and peritumoral edema can also be differentiated during navigation, again making the planning, identification and performance of resection planes easier.

In the present study, clean resection margins were achieved in 81.5% of all patients, placing our R0 rate in the midfield; recent studies have reported R0 rates ranging from 78% for colorectal carcinomas [44] to 85%, 86% to 94% for primary malignant tumors of the sacrum [1,30,45].

### 4.2. Survival Analysis

Among the factors known to determine the disease-specific survival of patients with sacral tumors, the underlying tumor biology, grading and stage of disease (presence of distant metastases), as well as the response to radio- or chemotherapy, seem to be most decisive. 

Although other studies showed slightly longer follow-ups of 33–53 months [44,46], the disease-specific survival rates (61–85%) [23,30,44,46] are comparable to those of the present study (77%, 27-month follow-up). The relatively large variance of progression and disease-free survival in our study and other studies [23,30,44,46] may be partly explained by the heterogeneity in underlying tumor biologies, as chordoma patients appear to have the best and sarcoma patients the worst outcome. 

The 1- and 2-year disease-specific survival rates for all patients in this study were 88% and 83%, respectively; for sarcoma patients, 86% and 71%; and for carcinoma patients, 89% and 62%, respectively. These are very much in line with the corresponding 1- and 2-year overall survival rates (28–88% and 16–63%, respectively) in the literature [1,13,47,48]. For sarcomas, similar 1- and 2-year survival rates of 62–92% and 63% have been reported [1,48]. These results, along with the relatively good disease-specific survival in patients with locally recurrent rectal carcinoma, underscore the notion that high and total sacrectomies with an achieved R0 resection in these patients are oncologically useful and contradict other studies that raised concerns regarding survival and high morbidity rates [49,50,51]. 

Many studies have shown that clear resection margins are the most decisive factors in predicting long-term survival in patients with advanced and recurring rectal cancer. They have further shown that R0 resections can be achieved in 78%, and a 5-year overall survival of 28–50% is expected [44]. In 89% of all carcinoma patients of our study, an R0 resection was obtained, although 77% of all resections were high- or total sacrectomies. These findings lend further support to the hypothesis that the completeness of a margin-free resection is more important than the level of sacrectomy. Out of the nine patients with locally recurrent rectal cancer, in eight patients, margin-free resection was achieved via high or total sacrectomy. The only one R1 resection was seen in the lateral margin of a hemisacrectomy, indicating that, regarding the attainment of R0 resection, lateral pelvic invasion seems more critical than the level of sacral involvement.

Sacrectomy studies explicitly comparing survival between sarcomas and carcinomas are rare, and, from an oncological point of view, performing a comparison of survival data is difficult due to the different tumor biology. Sacral involvement in the context of local carcinoma recurrence has an unfavorable prognosis. However, in a relevant number of these patients, local recurrence is restricted to the sacral/pelvic site without any metastatic disease. The current series included exactly those patients with locally recurrent rectal carcinoma that did not have any distant lesions, as verified by PET-CT. In these patients, the consequent extension of extralesional excision, i.e., high/total sacrectomy that was performed in two cases even en bloc with lumbar spine (en bloc L5 spondylectomy), reliably resulted in negative histopathologically resection margins.

As opposed to the very poor prognosis and discouraging results of sacrectomy in patients with sacral involvement due to metastatic disease [13], primary sacral tumors/sarcomas show progression-free survival rates in 20%–50% and 4%–75%, respectively [8,9,45,46,48,52]. Similar to the overall local recurrence rate of 28% of the present study, Ozdemir et al. reported a comparable rate of 23% in 12 sarcoma and 34 carcinoma patients, of whom only 23 patients underwent any surgical treatment [24]. Interestingly, the mean recurrence-free survival was 69 months (57%/11%/22% within the sarcoma/chordoma and carcinoma group), and metastatic disease developed in 52% after a mean interval of 46 months. A multivariate investigation of factors influencing disease-specific survival was not possible due to non-significant regression models. Due to small patient numbers, no significant factors could be identified for recurrence- and metastasis-free survival. Differences from the findings by Ozdemir [24] and other studies may also partly be explained by the comparably higher number (>50%) of patients with advanced disease (including two patients en bloc with L5) in the present study requiring total/high-sacrectomies. 

Independent factors influencing metastases after sacrectomy have hardly been studied. Clean surgical margins (R0 resection) do not only seem to lower the risk of local recurrence [1,15,23,30,46,53] but also contribute significantly to improved overall survival [1,7,15,23,30]. Wang et al. additionally found a significant difference in survival between high-grade and low-grade tumors [1]. In contrast, Hulen et al. did not find a significant effect of negative resection margins or pre/postoperative radiation [54]. Many studies show significantly prolonged disease-free and overall survival after the use of radiation [8,23,45,48,55,56]. Fujiwara et al. identified a minimum distance of 1.5 cm for adjuvant radiation for negative margins [55], while Sasikumar et al. identified a safety margin of at least 1 mm for wide local resections in carcinomas as a significant prognostic factor for disease-free and overall survival [44]. An alternative intralesional therapeutic strategy was described by Du et al., with only symptomatic/palliative curettage without filling showing a rapid reduction of pain, improvement of quality of life and a decreased complication rate, but also with very low survival rates, i.e., 41% and 22.5%, after 1 and 2 years, respectively, which they explained with a high proportion of fast-growing tumors [10]. According to Feiz-Erfan et al., the extent of resection (subtotal/total) in metastases does not influence overall survival, although there is a trend towards en bloc resection with regard to better local control [22]. Gosheger et al., however, found a significant difference in the extent of resection in patients with sacral/pelvic sarcoma [57]. 

In our study, patients’ age as an influencing factor (study age over 60 years) could not be identified as a significant influencing factor for survival. To the contrary, Samson et al. showed that higher age seems to have a trend towards the increased occurrence of recurrencies in sacral chordomas. Also, in sacral chordomas, Cheng et al. came to similar conclusions, as they showed increasing age and high sacral tumor localization to be associated with a higher recurrence rate. On the other hand, Varga et al. did not find an effect of high age on the recurrency rate but on overall survival in sacral chordoma patients. Regarding recurrence risk, they identified tumor size, previous tumor surgery on the same side and resection type as influencing factors [30]. Similarly, George et al. could not identify any influence of age on survival in chordoma patients [7], while Wulfften Palthe et al. were able to demonstrate tumor size to be an independent influencing factor for worse overall and recurrence-free survival. [56]. Similarly, Colangeli et al. were able to demonstrate an association between high resections to a lower overall survival and a higher recurrence risk [7,46].

### 4.3. Complications

Individual probability of risks for intra-/postoperative complications appears to be associated not only with tumor-specific factors (extent of pelvic/sacral/spinal invasion, vascularity, involvement of visceral organs and/or large vessels, etc.) and consecutive extent of resection (sacral transection level, intra- vs. extralesional excision and length of operative time) but is also closely linked to the mode of defect reconstruction (need for spinopelvic fixation and use of plastic reconstructive options). Furthermore, patient-related characteristics, i.e., previously treated with radio-/chemotherapy, and comorbidities are expected to have an additional impact on an individual risk profile.

Among the types of intraoperative complications, massive intraoperative blood loss with subsequent hemorrhagic shock, rectal perforation and pulmonary thromboembolism are reported to represent the most frequent causes, leading to intra- or early postoperative death in less than 3% [58]. In the present study, there was no intra- or very early postoperative death due to one of the above-mentioned reasons, as only one patient died of infection-induced multiorgan failure 5 months after surgery. The relatively low intraoperative complication rate in the present study may be due to the fact that all patients have been meticulously planned in terms of optimal local staging (MRI, PET and CT scans) to assess the extent of sacral/pelvic invasion and adherence to essential surrounding structures and make the decision for all posterior or sequential supine/prone approaches. In particular, the level and angle of sacral transection and lateral pelvic resection lines with sacrifice of sacral nerve roots have been determined on imaging and partly intraoperatively navigated. Furthermore, in all patients of that study with an expected sacral transection line above the level of sacroiliac joint (S3), combined sequential anterior–posterior approaches have been used with anterior sacral corticotomy/SI-joint dissection as a “predetermined breaking point”, making final osteotomy and resection from posterior much easier. The consequent use of a combined approach in high and total sacrectomies in our patients allowed for the maximum control and selective branch ligation of intrapelvic vessels, safe exposure and the dissection of lumbosacral nerve roots with careful ureterolysis under direct vision, thereby reaching complete clearance of the presacral space and minimizing risk for unintended injury during posterior approach. Zang et al. and Dubory et al. evaluated and compared the use of laparoscopy for the anterior approach in order to mobilize and ligate vascular structures and isolate the sacral tumor from surrounding structures. Blood loss and operation time have been reported to be significantly decreased [59,60].

We did not pursue that technique, as it does not allow for anterior corticotomy and lateral SI-joint dissection (predetermined breaking/transection point), and for the overwhelming majority of patients with combined approaches, the preparation of myocutaneous VRAM flap only possible via the first anterior approach had to be accomplished. Severe intraoperative bleeding resulting from arterial injury during sacral tumor resections has been proposed to be treated by either the direct transient cross-clamping (<15 min without heparinization) of the infrarenal aorta or the use of aortic balloon occlusion, both with low rate of complications. None of these techniques has been necessarily used in patients of the present study [61,62].

The most prevalent postoperative complications after sacrectomy represent surgical site infection, wound dehiscence, mechanical instrumentation failure, secondary cerebrospinal fluid leakage, urinary disorders and sensomotor dysfunction/fecal incontinence [1,58]. However, postoperative neurological deficits with either S1 dysfunction or loss of sphincter control following high or total sacrectomies are difficult to address in the current study, as they are an almost-invariable consequence of oncologically necessary sacral-nerve transections. The extent of neurological dysfunction, however, did not exceed the expected extent, as determined by the level of involved and transected nerve roots. All patients, except the one who died of multiorgan failure, have been ambulatory, partly using walking frames and sticks.

In particular, wound-healing disorders and wound dehiscence, including infections, are frequent and described in all current studies [1,17,19,54,63]. The described rate of infections following sacrectomy varies between 25 and 60% [1,44,58]. In the present study, it was higher, with 63%, including infections (37%) and wound-healing disorders (26%). If deep infections and superficial wound-healing disorders are analyzed separately, our study shows similar rates. Although wound complications do not always require revision surgery and revision rates vary between 30% and 55% [1,57], in our study, all patients with one or more complications were revised. 

Many studies investigated causative factors for the occurrence of complications. Wang et al. showed that neoadjuvant irradiation, rectal rupture, age <40 years, tumor size >10 cm and complex reconstruction were relevant influential factors [1]. Du et al. found, following intralesional curretages of sacral tumors, that patients without neoadjuvant radiation had a significantly lower risk of wound infection, and this risk substantially increased when surgical therapy was combined with neoadjuvant radiation. [10]. Li et al. and others also reported that neoadjuvant radiation was an independent risk factor for wound complications and revealed a significant difference in the incidence of (wound) complications following neoadjuvant radiation [13,17,19,63]. The findings of the present study, showing a trend towards more superficial wound-healing disorders when patients underwent neoadjuvant radiotherapy, underscore this notion of adverse influence of neoadjuvant radiation on wound infection. To the contrary, Gosheger et al. did not find any association with neoadjuvant irradiation or irradiation at all, regardless of timing [57]. On the other hand, Asaad et al. demonstrated a significant difference in complication rates as a function of sacral resection extent/transection level with patients who underwent high sacrectomy suffering from significantly more complications [63]. 

This relatively high rate of infectious complication in our study may occur due to and be explained by different reasons. First, nearly every patient had either neoadjuvant or adjuvant radiation or/and was treated before by chemotherapy/immunotherapy. Second, in comparison to other studies, the rate of high/hemi- or total sacrectomies in 20 of 27 patients (74%) requiring extensive spinopelvic hardware instrumentation in 13 patients (48%) is comparably high. Although the level of sacral transection, use of spinopelvic instrumentation and previous radio-/chemotherapy are expected to increase operation times and infection rate, we could demonstrate a marked correlation only between implant-associated complications (screw loosening) and previous neoadjuvant radiotherapy, possibly indicating impaired ingrowth and anchorage of instrumentation in irradiated bone. However, neither the use of navigation, spinopelvic fixation, omentoplasty nor neoadjuvant radiotherapy, or the level of sacral transection, significantly influenced the rate of infection in our patient cohort. This lack of correlation may result from either the small number of cases or other causative factors and confounders that have not been analyzed and found in this study. Interestingly, although involving a more complexing reconstruction, Bedermann et al. also were able to demonstrate a decreased infection rate and blood loss following total sacrectomy in patients who received spine column fixation compared to those with only posterior pelvic ring instrumentation, lending support to the hypothesis that degree of stability may also partly influence incidence of infection [9].

Precise differential indications for dead-space filling and plastic lumbosacral or perineal defect coverage strictly depend on the remaining tissue, the vascular situation after resection, the approach and a neoadjuvant radiation [17]. In our study, only primary reconstruction methods, omentoplasty or vertical rectus abdominal muscle (VRAM) flaps were used. However, the use of VRAM-flap transfer was associated with a significantly decreased rate of infection, indicating the protective effect of recruiting healthy, well-vascularized tissue into the region that has been irradiated and potentially contaminated. In addition, by interposing healthy flap skin between irradiated perineal wound edges, wound healing is promoted. Since reliable conclusions on flap transfer-induced influence on wound-healing disorders and infections are only possible with correspondingly higher case numbers, Sasikumar et al. could not make any statement on the effectiveness but described the protective effect of flaps to avoid perineal infections [44]. Kim et al. reported an association with increased revision incisions and infections and the VRAM-flap [17] but still consider the VRAM flap as the gold standard for soft tissue reconstruction after total sacrectomy, as it is effective at filling the pelvic dead space and also prevents hematoma, infection and peritoneal herniation [17]. Notably, in their study, Asaad et al. showed an overall high complication rate of 50% with VRAM flaps [63] that, however, could not be confirmed in later studies with higher patient numbers [64]. Although VRAM-flap transfer may be difficult in presence of previous transverse laparotomy or bilateral stomas, the technique seems to be superior when compared to gluteal muscle flaps, which may be partly devascularized due to sacrifice of inferior–superior gluteal artery or internal iliac vessel ligation.

The finding of significantly fewer wound-healing disorders with the use of preoperative stoma strengthens the concept of prevention of infectious complications via the use of protective ilio-/colostomy. Regarding the use of navigation, no complications related to navigation have been described so far [11,40], and this is also the same in our study.

Complete hemisacrectomy or transection of the sacrum above S2, leaving only a small and critically stable sacroiliac (SI) joint intact, requires reconstruction in order to maintain or restore pelvic-ring stability and allow for spinopelvic load transmission. For this, various methods, including spinopelvic fixations, allograft bone fusions, sacroiliac screws or even 3D-printed sacrum, have been described [16]. Currently, different types of spinopelvic reconstructions using lumbar pedicle and iliac screws and—depending on the width of the remaining sacral corridor above and below S1 foramina—iliosacral screw/bar fixation are the favored reconstructive methods. They can be combined with the use of bone grafts for continuity restoration [15,65,66,67]. In our study, spinopelvic fixations were performed in 48% of cases, primarily after total or hemisacrectomy. Independently from the type of instrumentation and hardware used for posterior fixation but also in view of the fact that bony fusion can only rarely expected, all methods are inherently associated with complications. They typically result from painful soft tissue irritation by prominent screws, cross-connectors and rods or in the long-term follow-up consecutive fatigue implant failure with loosening or dislocation [1,15]. Since we report only a mid-term follow-up period in a moderate number of patients, only a few implant-associated postoperative complications were recorded. Nevertheless, we usually use a polyaxial screw with the maximum possible thickness and a cement augmentation that are fixed to U-shaped double-rod (6 mm, chrome–vanadium) reconstructions with side-to-side and cross-connectors, as they are reported to offer optimum stability and ideal preconditions for bone fusion [68].

In addition, in total sacrectomy patients and those in whom L5 is additionally resected in terms of an en bloc spondylosacrectomy (two patients in our study), we try to shorten the distance between the lumbar spine and the remaining posterior pelvic-ring structures/iliac wings via axial spinopelvic compression along the rods in order facilitate bony fusion and ingrowth of bone graft and reduce the working length of the iliolumbar construct.

However, due to the high rate of complications, future clinical trials should be designed that compare the oncological outcome, clinical benefit and complication profile of partial or total en bloc sacrectomy to alternative therapy methods, especially in neoadjuvant radiotherapy of primary sacral bone tumors. 

For chordoma and also chondrosarcoma, there are promising reports with dose-escalating neoadjuvant stereotactic body radiation therapy (SBRT) that indicate a favorable safety profile and efficacy without leading to excessive surgical risks and increasing the likelihood for successful en bloc resection [69]. Moreover, recent reports also suggest that high-dose spinal single-fraction radiosurgery (SRS) offers the chance for durable radiological control and effective symptom relief with acceptable toxicity in patients with primary chordomas as either a definitive or adjuvant therapy [69].

Carbon ion therapy (CIRT), classified as high linear energy-transfer radiation, has emerged as another promising modality in the treatment of various types of cancers, including pelvic tumors [70], and is expected to be effective for even photon-resistant tumors [71]. Shiba et al. reported on the use of CIRT for recurrent rectal cancer. They report similar 2-year overall survival and recurrence- and progression-free survival rates after CIRT of 100%, 83% and 29%, respectively. Moreover, Jamada et al. were able to show 5-year survival of 59% with local control rates of 88% following CIRT for recurrent rectal cancer [72,73,74]. However, the data on CIRT for sarcomas remain poor. For inoperable chondrosarcomas, Imai et al. reported the results of CIRT with local control, overall and disease-free survival of 53%, 53% and 34%, respectively. However, subsequent surgical revisions due to pathological fractures have been shown to be necessary in 7% [75], concluding that CIRT could represent a treatment option for irresectable chondrosarcomas. In a multicenter study involving 911 patients, Yolcu et al. investigated the efficacy of CIRT in chordoma patients who were classified as non-resectable. There was no difference in the 5-year overall survival, local recurrence-free survival and metastasis-free survival compared to patients with margin-free en bloc resection. Patients with CIRT even showed significantly less peripheral motor neuropathy. Compared to R1 resection without adjuvant radiotherapy and compared to primary radiotherapy, patients with CIRT showed significantly longer overall survival [76]. Therefore, CIRT may offer itself to be a promising alternative treatment of pelvic chordomas, particularly if surgical intervention is not feasible or carries a high risk of complications, or it can function as an adjuvant method in R1 situations [76]. 

However, longer follow-up and higher patient numbers are needed in prospective studies for assessing efficacy in terms of local and systemic tumor control. 

## 5. Conclusions

In our study, we were able to report on surgical and oncologic outcomes after partial or total sacrectomy for primary malignant bone tumors and locally recurrent rectal cancer with a margin-free en bloc resection rate of 81% and median overall survival of 70 months. While there was a significant difference in progression-free and metastasis-free survival between sarcoma, chordoma and carcinoma patients, the occurrence of metastases was significantly associated with shorter survival, regardless of the tumor entity. Resection of sacral tumors is extraordinarily challenging, as it is associated with a high complication profile and should follow an oncosurgical multidisciplinary team approach, involving orthopedic, visceral and vascular surgical expertise. For complications, no independently influencing factors could be identified, except for a protective effect of VRAM-flap transfer and colostomy on postoperative infection rates. For patients with sacral involvement due to locally recurrent rectal cancer and who usually had radiation as part of their treatment for primary tumor, total en bloc resection offers the best and only chance for local control and long-term prognosis. For patients with primary bone tumors, excellent oncological outcome and increased morbidity of en bloc sacrectomy have to be compared to promising results of either dose-escalating neoadjuvant stereotactic body radiation therapy (SBRT) prior to en bloc resection or high-dose spinal single-fraction radiosurgery or carbon ion radiotherapy as definitive or adjuvant treatment. Therefore, prospective multicenter studies with a longer follow-up and higher patient numbers are needed.

## Figures and Tables

**Table 1:** Clinical characteristics.

				All *n* = 27 (%)	Sarcoma *n* = 8 (29.6%)	Chordoma *n* = 10 (37.0%)	Carcinoma *n* = 9 (33.3%)	*p* (χ^2^-Test)
**Sex**	Female			13 (48)	6 (30)	3 (30)	4 (44)	0.159
	Male			14 (52)	2 (25)	7 (70)	5 (56)	
**Histology**	Chordoma			10 (37)	-	10 (100)	-	-
	Osteosarcoma			3 (11)	3 (37.5)	-	-	
	Pleomorphic sarcoma			2 (7)	2 (25)	-	-	
	Chondrosarcoma			1 (4)	1 (12.5)	-	-	
	Rhabdomyosarcoma			1 (4)	1 (12.5)	-	-	
	Myxoid liposarcoma			1 (4)	1 (12.5)	-	-	
	Colorectal carcinoma			9 (33)	-	-	9 (100.0)	
**Appearance**	Primary tumor			18 (67)	8 (100)	10 (100)	-	-
	Local recurrence			9 (33)	-	-	9 (100)	
**Multimodal** **therapy**	Neoadj. chemotherapy			13 (48)	5 (62.5)	-	8 (89)	0.004 *
	Neoadj. immunotherapy			6 (22)	2 (25)	1 (10)	3 (33)	0.367
	Neoadj. radiation			8 (30)	2 (25)	1 (10)	5 (56)	0.072
	Adj. radiation			5 (18.5)	1 (12.5)	4 (40)	-	0.071
**Sacrectomy level**	Total			9 (33)	2 (25)	3 (30)	4 (44)	0.670
	Partial high		S1	3 (11)	-	-	3 (33)	
			S2	2 (7.5)	-	2 (20)	-	
	Partial low		S3	4 (15)	-	4 (40)	-	
			S4	1 (4)	-	1 (10)	-	
	Hemi total			6 (22)	4 (50)	-	2 (22)	
	Hemi partial (S1-S3)			2 (7.5)	2 (20)	-	-	
**Approach**	Posterior			9 (33)	3 (37.5)	6 (60)	-	0.021 *
	Anterior–posterior			18 (67)	5 (62.5)	4 (40)	9 (100)	
**Additional**	Intraop. 3D navigation			12 (44)	4 (50)	3 (30)	5 (56)	0.498
	Spinopelvic fixation			13 (48)	6 (75)	3 (30)	4 (44)	0.159
	VRAM flap			10 (37)	2 (25)	1 (10)	7 (78)	0.007 *
	Omentum majus transfer			20 (74)	5 (62.5)	6 (60)	9 (100)	0.094
	Stoma			21 (78)	4 (50)	9 (90)	8 (89)	0.079
**Resection status**	R0			22 (81.5)	6 (75)	8 (80)	8 (89)	-
	R1			4 (15)	2 (25)	2 (20)	-	
	Rx			1 (3.5)	-	-	1 (11)	

* Significant results in the 95% confidence interval.

**Table 2:** Outcome data.

Outcome		All *n* = 27 (%)	Sarcoma *n* = 8 (30%)	Chordoma *n* = 10 (37%)	Carcinoma *n* = 9 (33%)
**Status of last follow-up**	NED ^1^	13 (48)	2 (25)	8 (80)	3 (33.3)
	AWD ^1^	5 (19)	2 (25)	-	3 (33.3)
	DOD ^1^	6 (22)	3 (37.5)	-	3 (33.3)
	DOC ^1^	1 (4)	-	1 (10)	-
	LTF ^1^	2 (7)	1 (12.5)	1 (10)	-
**Distant metastasis** ^2^	Yes	13 (52)	6 (86)	1 (11)	6 (67)
	No	12 (48)	1 (14)	8 (89)	3 (33)
**Local recurrence** ^2^	Yes	7 (28)	4 (57)	1 (11)	2 (22)
	No	18 (72)	3 (43)	8 (89)	7 (78)

^1^ NED: alive with no evidence of disease; AWD: alive with disease; DOD: dead of disease; DOC: dead of other causes; LTF: lost to follow-up. ^2^ Without lost-to-follow-up patients, *n* = 25.

**Table 3:** Results of the log-rank test according to the Kaplan–Meier method.

	Disease-Specific Survival *p* =	Progression-Free Survival *p* =	Metastasis-Free Survival *p* =	Local Recurrence-Free Survival *p* =
**Age ≥ 60 years**	0.702	0.104	0.108	0.381
**Sarcoma/chordoma/carcinoma**	0.106	0.003 * (Sar/Chor/Ca: 10/84/24 mon.)	0.003 * (Sar/Chor/Ca: 15/84/24 mon.)	0.024 * (Sar/Chor/Ca: 16/84/74 mon.)
**Neoadj. chemo-/immunotherapy**	0.087	0.017 * (yes/no: 22/70 mon.)	0.014 * (yes/no: 22/70 mon.)	0.179
**Neoadj. radiation**	0.962	0.706	0.868	0.328
**Adj. radiation**	0.148	0.071	0.065	0.361
**Intraop. 3D navigation**	0.819	0.627	0.548	0.491
**Partial/total sacrectomy**	0.735	0.134	0.256	0.167
**Approach (post./ant.–post.)**	0.920	0.104	0.124	0.050 * (ant.-post. < post.)
**Spinopelvic fixation**	0.296	0.010 * (yes/no: 18/68 mon.)	0.020 * (yes/no: 20/68 mon.)	0.006 * (yes/no: 41/87 mon.)
**Omentum transfer**	0.769	0.210	0.239	0.077
**R0 resection**	0.928	0.887	0.771	0.161
**Any complication**	0.780	0.710	0.692	0.674
**Infection**	0.814	0.148	0.349	0.092
**Metastases in FU**	0.017 * (yes < no)	<0.001 * (yes < no)	-	0.002 * (yes < no)
**Recurrence in FU**	0.899	0.044 * (yes/no: 13/62 mon.)	0.066	-

* Significant results in the 95% confidence interval.

**Table 4:** Complications.

			All Patients *n* = 27 (%)	Posterior Approach *n* = 9 (%)	Anterior–Posterior Approach *n* = 18 (%)
**All complications**			22 (81.5)	7 (78)	15 (83)
**Wound-healing disorder/infection**	All		17 (63)	5 (56)	12 (67)
	Deep infection		10 (37)	2 (22)	8 (44)
	Wound-healing disorder		7 (26)	3 (33)	7 (39)
	With sepsis		3 (11)	-	3 (17)
**Hematoma/bleeding**			6 (22)	2 (22)	4 (22)
**Implant-associated**	All (8 complications)		6 (22)	2 (22)	4 (22)
	Rod breakage	*n* =	2 (7)	-	2 (11)
	Rod dislocation	*n* =	2 (7)	1 (11)	1 (6)
	Screw breakage	*n* =	2 (7)	1 (11)	1 (6)
	Screw malposition	*n* =	1 (4)	-	1 (6)
	Screw connector loosening	*n* =	1 (4)	1 (11)	-
**Urological**	Urinary tract infection		3 (11)	-	3 (17)
**Thromboembolic**	All		2 (7)	-	2 (11)
	4-level thrombosis		1 (4)	-	1 (6)
	Pulmonary artery embolism		1 (4)	-	1 (6)
**Visceral surgical**	All		2 (7)	1 (11)	1 (6)
	Paralytic ileus		1 (4)	1 (11)	-
	Ischiorectal hernia		1 (4)	-	1 (5.6)
**Fracture**	Insufficiency fracture SWK1		2 (7)	-	2 (11.1)
**Pneumonia**			1 (4)	-	1 (5.6)

**Table 5:** Univariate regression analysis.

	Any Complication *p* (χ^2^-Test) =	Deep Infection *p* (χ^2^-Test) =	Wound-Healing Disorder *p* (χ^2^-Test) =
**Age ≥ 60 years**	0.686	0.516	0.745
**Neoadj. chemo-/immunotherapy**	0.825	0.656	0.326
**Neoadj. radiation**	0.280	0.974	0.064
**Adj. radiation**	0.925	0.879	0.738
**Intraop. 3D Navigation**	0.825	0.516	0.580
**Sacrectomy level**	0.488	0.491	0.364
**Approach**	0.726	0.260	0.535
**Spinopelvic fixation**	0.686	0.883	0.745
**VRAM flap**	0.879	0.042 *	0.065
**Omentum majus transfer**	0.738	0.148	0.235
**Stoma**	0.555	0.241	0.010 *

* Significant results in the 95% confidence interval.

## Data Availability

All data obtained and analyzed for this clinical study are available from the corresponding author upon reasonable request.
